# Characteristics of *Panax ginseng* Cultivars in Korea and China

**DOI:** 10.3390/molecules25112635

**Published:** 2020-06-05

**Authors:** Hao Zhang, Suleman Abid, Jong Chan Ahn, Ramya Mathiyalagan, Yu-Jin Kim, Deok-Chun Yang, Yingping Wang

**Affiliations:** 1 State-Local Joint Engineering Research Center of Ginseng Breeding and Application, Jilin Agricultural University, Changchun 130118, China; zhanghaoscience@163.com; 2 Institute of Special Wild Economic Animals and Plants, Chinese Academy of Agricultural Sciences, Changchun 130112, China; 3 Graduate School of Biotechnology, College of Life Sciences, Kyung Hee University, Yongin si, Gyeonggi do 17104, Korea; sulmanabid1994@gmail.com (S.A.); jongchanahn7@gmail.com (J.C.A.); ramyabinfo@gmail.com (R.M.); yujinkim@khu.ac.kr (Y.-J.K.)

**Keywords:** *Panax ginseng*, ginseng species, *Panax ginseng* cultivars, Chunpoong, Jilin Huangguo Renshen

## Abstract

Ginseng (*Panax ginseng* Meyer) is one of the most important medicinal herbs in Asia. Its pharmacological activity comes from ginsenosides, and its roots are produced commercially for traditional and Oriental medicine. Though 17 *Panax* species are available around the world, there was a need to develop cultivars adapted to different climatic conditions and resistant to various diseases while still producing high-quality, high-yield roots. Thus, 12 and 9 commercial *P. ginseng* cultivars have been registered in South Korea and China, respectively. Those varieties show superiority to local landraces. For example, Chunpoong is more highly resistant to rusty rot disease than the local Jakyungjong landrace and has a good root shape; it is highly cultivated to produce red ginseng. The Chinese cultivar Jilin Huangguo Renshen has higher ginsenoside content than its local landraces. This review provides information about *P. ginseng* cultivars and offers directions for future research, such as intra- and interspecific hybridization.

## 1. Introduction

Ginseng, which belongs to the genus *Panax* and family Araliaceae, is widely used in East Asia as an herbal medicinal plant, as it has excellent medicinal properties [1]. Particularly in Korea, China, and Japan, ginseng is considered to be the most important of all medicinal herbs. For the past 2000 years, people from those places have used ginseng root and its extract as a stimulant to relieve stress and fatigue, strengthen the body and mind, prevent aging, and increase vigor [2,3]. The *Panax* genus consists of 17 species, but *P. ginseng* (Asian or Korean ginseng), *P. quinquefolius* (American ginseng), and *P. notoginseng* (Chinese ginseng) are the species most commonly used as a functional food and medicine [4]. The genus *Panax*, first used by the Russian botanist, Carl A Meyer [5], is derived from the Greek *pan*, meaning “all”, and *axos*, meaning “medicine”, indicating that ginseng is a cure for all diseases [6]. Due to the resemblance between the ginseng root and the human shape, the English name “ginseng” was introduced from the Chinese word “renshen” [7]. 

According to the archaeological evidence, pharmaceutical use of ginseng originated in the Paleolithic period more than 60,000 years ago. The world’s oldest pharmacopeia of medicinal herbs and plants, *Shennong’s Herbal Classic* [8], organizes herbs into three classes by their degree of toxicity. Ginseng was deemed to be a nontoxic medicinal plant and recommended for regular use to increase energy [9]. For a long time, human beings have been using medicinal plants as beverages, nutrients, dyes, cosmetics, and medicines to improve their quality of life and maintain good health [8]. 

Around the world, especially in Asia, many people still depend on herbal medicines to treat and prevent different health conditions. The World Health Organization reported that 80% of the worldwide population depends on herbal medicines as supplemental or alternative medicines [10]. According to the yin and yang theory, ginseng, “the king of all herbs” plays a vital role in the pharmacopeia and is valued for its significant therapeutic properties [11], many of which have been validated, including its anti-inflammatory [12], antioxidative [13], anti-obesity [14], anti-allergic [15], antihypertensive [16], memory improvement [17], sexual potentiation [18,19], anti-diabetic [20], and antitumor [21] properties. Ginseng also modulates metabolism, immune functions, and blood pressure [22]. The mechanism of ginseng’s action remained unidentified until secondary metabolites such as ginsenosides were isolated in 1963. Since then, much effort has been focused on evaluating the mechanism and function of each ginsenoside because different species of ginseng vary in ginsenoside content. Furthermore, a single species cultivated in different locations can have pharmacological differences. Ginseng has many ginsenosides, each of which can have many pharmacological effects. Furthermore, non-ginsenoside bioactive components in ginseng also have pharmacological properties. Therefore, it is not astonishing that the whole activity of the herb is complex and still needs more exploration [22]. Nonetheless, most *Panax* species are morphologically similar, though their origin is not completely understood. Cytogenetic data have been used to explore their origin distribution, but still more research is needed to fully understand the phylogenetic relationships among *Panax* species [23]. Also, plant secondary metabolites are often used for medicinal purposes, and ginseng has different kinds of secondary metabolites (Table 1). 

Customers need to understand ginseng species and cultivars so they can make correct choices for medicine. *P. ginseng* (Asian or Korean ginseng) is used as a restorative medicine [24]. *P. quinquefolius* (American ginseng) is also used to treat thirst, fatigue, dryness of the mouth, the respiratory tract, and irritability [25]. Asian ginseng and American ginseng have different types of ginsenosides, including Rf and F11, respectively, that are missing from pseudo-ginseng. Other ginseng species are *P. notoginseng* (Sanchi ginseng) [26], *P. japonicus* (Japanese ginseng) [27], *P. vietnamensis* (Vietnam ginseng) [28], *P. pseudoginseng*, *P. assamicus*, *P. shangianus*, *P. variabilis*, *P. major Ting*, *P. omeiensis*, *P. sinensis*, *P. stipuleanatus*, *P. trifolius*, *P. wangianus*, and *P. zingiberensis* [29] (Table 2). Generally, people only use good brands or famous species for pharmaceutical purposes.

## 2. Phytochemistry of *Panax ginseng*

**Figure 1:**
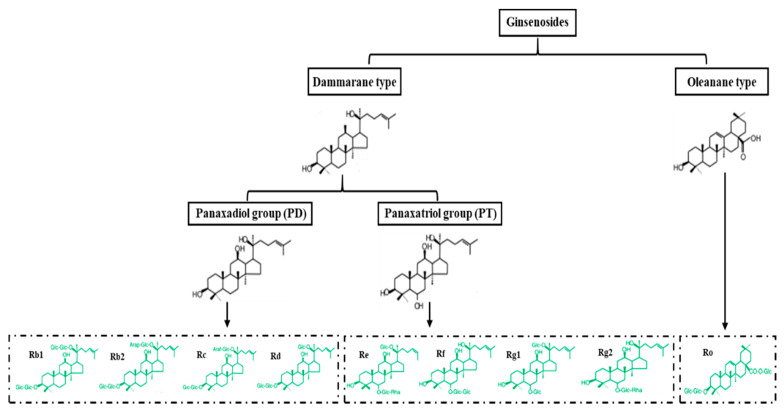
Classification of ginsenosides by chemical structure.

Ginseng produces various kinds of secondary metabolites that have important biological purposes. The ability to perform in vivo combinative chemistry by evolving the genes needed for different secondary metabolite biosynthetic pathways is likely to have been difficult in the diversification of different plants. Terpenes are secondary metabolites found in various kinds of plant-based products [61]. Numerous secondary metabolites, including saponins, polysaccharides, flavonoids, and amino acids have been found in *Panax* plants. Saponins, also known as ginsenosides, are the main bioactive constituents of the pharmacological efficacy in natural medicines derived from *Panax* species. The well-known ginsenosides have different chemical structures [62], with different medicinal properties as shown in Table 3. In ginseng, the most unique compounds are the ginsenosides (panaxosides), which are present in the form of saponins, including the triterpene saponins [63]. Ginsenosides are divided into two groups (Figure 1): dammarane and oleanane, which have four- and five-ring carbon skeletons, respectively [64]. Ginsenosides are generally represented as “Rx”, where the “R” stands for the root and the “x” defines the chromatographic polarity [5]. Dammarane groups are further divided into protopanaxadiol (PPD) groups Rb1, Rb2, Rc, and Rd, and protopanaxatriol (PPT) groups Re, Rf, Rg1, and Rg2 (Figure 1) with different sugar moieties that are attached to the C-3 and C-20 positions or C-6 and C-20 positions, respectively. At least 289 ginseng saponins were reported from eleven *Panax* species at the end of the year 2012 [62]. The presence of these compounds increases the value of ginseng in the medicinal field worldwide. Furthermore, ginsenosides yields vary in ginseng species due to the different cultivation methods and different chemical structures [2,65,66]. 

## 3. Breeding Method for the Development of Ginseng Cultivars

Ginseng (*P. ginseng*) is a perennial and self-fertilized plant. Ginseng breeding is difficult because it takes four years of growth for seed production, and even after four years, ginseng produces very few seeds [84]. Different cultivars have been developed from *P. ginseng* species through pedigree breeding programs to select good characteristics from local landrace populations such as Hwangsook, Chungkyung, and Jakyung. Initially, local landraces were differentiated and selected by morphological characteristics, including stem and berry colors. Cultivars with good characteristics were chosen by judging the characteristics of individual plants in a farmer’s field, and then inbreeding was done by pedigree in Korea [85]. In the pedigree method, good individual plants are segregated for generations, and their progeny are checked in succeeding generations. Later, the pure line selection method was used to develop ginseng cultivars from *P. ginseng*. Pure line selection can use two different methods, line separation and propagation on the one hand, and hybridization (crossbreeding, reciprocal crosses, and mutant breeding) on the other. The line separation and propagation method uses three steps. In the line fixing step, we check the plants’ resistance to disease, salt, light, and temperature. The second step is the product performance test, and the final step tests regional adaptability in field trials. This process requires 5–6 generations and 20–24 years to develop a new cultivar. For the hybridization method, good individual plants are selected for breeding; this method also requires 5–6 generations and 20–24 years to develop a new cultivar [86,87].

## 4. *Panax ginseng* Cultivars in Korea 

### 4.1. Chunpoong

**Figure 2:**
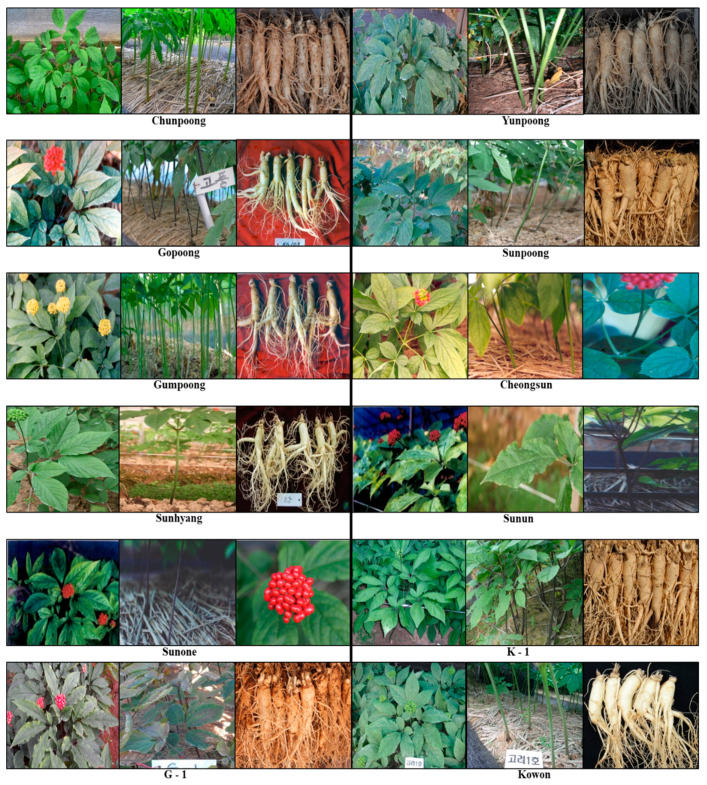
*P. ginseng* cultivars in Korea. All cultivars have superiority over the parents produced by pure-line selection breeding methods.

Chunpoong is a *P. ginseng* cultivar developed by Woo-Saeng Kwon. To develop this new cultivar, many different individual ginseng plants were screened for high yield and good quality in a farmer’s fields in 1972. Many lines were selected, and a favorable one, 7259-3-1, was produced at the Korea Ginseng & Tobacco Research Institute by comparative cultivation using the pure line separation method. After it passed yield and adaptation trials, it was commercialized as Chunpoong [84]. Chunpoong has a high yield, good quality, and high resistance to ginseng rust rot disease. It has a green stem with light violet and orange-yellow flowers and fruits 37 days later than the local landrace, Jakyungjong. Chunpoong has an average stem length, stem diameter, leaflet length, leaflet width, palmate cleaves, and number of leaflets of 38.5 cm, 7.4 mm, 15.9 cm, 6.3 cm, 5.6, and 22.7, respectively. The below-ground sections of a four-year-old plant have an average main root length, diameter, and root weight of 8.3 cm, 27.0 mm, and 61.0 g, respectively. The taproot (main root) of Chunpoong is longer than that of the local landrace, and the root yield of Chunpoong is 9% greater than that of Jakyungjong. In red ginseng quality, the rates of Chun-Jeesam (“Chun” and “Jee” stand for first- and second-grade root ginseng, respectively) were 22.3% and 9.4% for Chunpoong and Jakyungjong, respectively. Due to its good root shape, it is widely used to produce red ginseng. Chunpoong has high market demand at the commercial level and is the most-grown cultivar of *P. ginseng* in South Korea (Figure 2) [88].

### 4.2. Yunpoong

Yunpoong was designated as KG-102 during its development, and then it was registered as the new cultivar Yunpoong by the Korea Ginseng & Tobacco Research Institute. It was developed using comparative cultivation of several lines selected with pure line separation from local landraces. In its mature stage, we can easily differentiate the Yunpoong cultivar. The flowering date of Yunpoong is earlier than that of other *P. ginseng* cultivars, and it forms more double ovary–type flowers. It has variant features and high yield, is widely cultivated, and has good root weight and a very thick root, which is helpful to the mass production of ginseng. The ratio of taproot length to the diameter of the taproot length is less in Yunpoong than in Jakyungjong, but the four-year-old root yield of Yunpoong is 27.3% more than that of Jakyungjong. This cultivar is widely used for mass production (Figure 2) [89].

### 4.3. Gopoong

Gopoong is also a *P. ginseng* cultivar introduced by Woo-Saeng Kwon using the same procedures just described for Chunpoong and Yunpoong. A promising line, 680-83-4, was named KG-103 and then registered with the Korea Seed & Variety Service (http://www.seed.go.kr) as the new cultivar Gopoong at the commercial level. It has a unique phenotype: a reddish or dark violet stem, dark red fruit, high saponin content, good looking root shape, and an inverted triangle shape of berries clusters, unlike other cultivars of *P. ginseng*. Gopoong shows a purple color around the stems and along the length of the petiole and peduncle. The four-year-old taproot of Gopoong is long, and the root yield is 4.5% higher than that of Jakyungjong. It also has red ginseng quality, with excellent grades of Chun-Jeesam. The ratio of red ginseng quality is 16.6% in Gopoong and 9.4% in Jakyungjong. As shown by this ratio, Gopoong is a superior *P. ginseng* cultivar with good quality for the production of red ginseng products (Figure 2) [90,91].

### 4.4. Sunpoong

*P. ginseng* cultivars have been developed to improve the root quality and yield. The characteristics of the Chunpoong cultivar are better than those of the local landraces. For example, its production of heaven- and earth-grade ginseng is 613% higher than that of the local variants, but its average root weight is less. Thus, the average grade of raw Chunpoong ginseng is lower than that of the local variants. Therefore, it was essential to breed a variant with a high rate of heaven and earth grades and heavy root weight. A superior line, 7224-1-1, was selected for testing and breeding due to the long length of its peduncle, and it was then registered as KG104 by the Korean Ginseng Corporation. After checking its productivity in adaptation experiments from 1981 to 1984, its three-year-old ginseng root weight was 15.4% higher than that of Chunpoong, and the new cultivar was approved as Sunpoong. Sunpoong has an early germination time. It has purple stems, a round and simple kind of inflorescence and berries, and stolon roots. Its berries and autumn leaves are red, its roots have a creamy color, and its leaflets are wide and elliptical. Sunpoong is a high yielding cultivar in terms of its average root weight. Its 1st- and 2nd-grade ginseng root percentages are higher than those of Yunpoong, 20.9% and 9.4%, respectively (Figure 2) [91,92].

### 4.5. Gumpoong

Gumpoong is also an excellent cultivar of *P. ginseng*, with good root shape and high yield that make it suitable for the production of red ginseng. It originated from the landrace Hwangsook [93]. Gumpoong was discovered in Gaepung-gun, Gyeonggi-do, in 1926 and in Jangdan-gun, Gyeonggi-do, in 1928. It has green stems, and its mature fruits are yellow, so it is also called the yellow berry cultivar. Studies have been conducted on the aerial part during flowering to characterize Gumpoong in terms of its relative elongation, saponin content, chlorophyll content, mineral content, and the photosynthetic content of its leaves and roots. Gumpoong was selected for its high resistance to rust roots in the Punggi area in 1979. Its characteristics were examined from 1979 to 1992, and then it was registered as the approved cultivar Gumpoong in 1996. Regarding its qualitative characteristics, Gumpoong does not have purple (or anthocyanin) in the stems, and its berries and inflorescence are simple and round. Its stem color is green, with yellow berries, yellow autumn leaves, a creamy root color, and broad elliptical leaflets. Its average stem length, stem diameter, leaflet length, leaflet width, palmate cleaves, and number of leaflets are 35.5 cm, 7.6 mm, 16.3 cm, 7.1 cm, 5.5, and 23.6, respectively, and its three-year-old main root average length, diameter, and root weight are 7.6 cm, 26.8 mm, and 70.2 g, respectively. Its average rates of heaven- and earth-grade ginseng are 21.2% and 12.8%, respectively (Figure 2) [85,91]. 

### 4.6. Cheongsun

Cheongsun was first introduced in Jangdan-gun, Gyeonggi-do, in 1927 as a green-stem variant with red berries. The same variant was produced at the Ginseng and Tobacco Research Institute, Jeungpyeong in Chungcheongbuk-do, South Korea in 1978. Studies with different entities were conducted for stem color distribution in 1988. Most entities showed green stems and red berries, but some showed green and purple stems in a 3:1 ratio. No significant differences were found in respiration or photosynthesis between the green-stem variant, the violet-stem variant, and the yellow berry variant. The susceptibility to root rot, cracking, and rusty roots were studied, and the yellow-berry variant and violet-stem variant did not differ significantly. In 1978, the green-stem variant, with green stems, peduncle, and petiole and red berries, was selected at the Ginseng & Tobacco Research Institute, Jeungpyeong. After breeding trials, the green-stem variant was registered as a new variety, Cheongsun, in 1991, and adaptation trials were completed in 1999. The qualitative features of Cheongsun are no purple in the stems, round and simple berries and inflorescence, and stolon roots. Cheongsun has red autumn leaves, broad elliptical leaflets, and a creamy root color. The average stem length, stem diameter, leaf length, leaf width, palmate cleaves, and number of leaflets of Cheongsun are 32.2 cm, 7.0 mm, 14.7 cm, 6.7 cm, 4.8, and 23.8, respectively. The three-year-old main root average length, diameter, and root weight are 8.6 cm, 25.4 mm, and 73.7 g, respectively. For the quality of red ginseng, the average rates of heaven- and earth-grade ginseng are 14.6% and 7.2%, respectively (Figure 2) [91,93,94]. 

### 4.7. Sunhyang

The Sunhyang *P. ginseng* cultivar was bred from Chunpoong (for its good morphological characteristics), Yunpoong (for its excellent yield), and Gopoong (for its high saponin content). Over time, more ginseng cultivars are being introduced because people are interested in using ginseng variants to promote a healthy lifestyle. Studies have been done for red ginseng taste, aromatic ingredients, and the identification of ginseng volatile compounds by gas chromatography and mass spectrometry. Entity 78093 was used for breeding from 1979 to 1981 and then named KG110. During those studies, it was realized that this variant had a high content of Aroma-1, which led to its approval as a new cultivar, Sunhyang. The aerial sections of Sunhyang are characterized by purple stems rich in anthocyanin, inflorescence, and simple, round berries. Sunhyang has stolon roots, red berries, red autumn leaves, broad elliptical leaflets, and cream-colored roots. The average stem length, stem diameter, leaf length, leaf width, palmate cleaves, and number of leaflets are 39.6 cm, 6.6 mm, 14.9 cm, 6.5 cm, 4.8, and 23.8, respectively. The main root average length is 7.4 cm, the average diameter of the main root is 29.0 mm, and the average root weight is 78.8 g. For the quality of red ginseng, the average rates of heaven- and earth-grade ginseng are 13.3% and 14.1%, respectively (Figure 2) [91,94,95].

### 4.8. Sunun

Self-fertilization occurs in ginseng, and breeding procedures mostly depend on the morphological characteristics of the aerial and below-ground sections, due to its limited genetic resources. Thus, different cultivars have been developed by selecting for good morphological appearance. A variant was selected with strong blistering on the leaflet surface in 1985, and then in 1989, a purple-stem population of that variant was approved as the Sunun cultivar. The aerial part of Sunun has purple stems, inflorescence, round and simple berries, and stolon roots. The berries and autumn leaves are red. The leaflets are broad and elliptical, and the root color is cream. The average stem length, stem diameter, leaf length, leaf width, palmate cleaves, and number of leaflets are 29.0 cm, 6.9 mm, 16.2 cm, 7.0 cm, 4.5, and 24.3 respectively. The three-year-old main root average length, diameter, and root weight are 8.2 cm, 26.5 mm, and 67.8 g, respectively. For the quality of red ginseng, the average rates of heaven- and earth-grade ginseng are 16.4% and 18.0%, respectively (Figure 2) [91].

### 4.9. Sunone

Root rot is one of the major diseases in ginseng and causes yield reduction in ginseng cultivation. Root rot disease also disturbs the continuous cropping of ginseng. To produce a rot-resistant root ginseng cultivar, several resistant variants were selected in 1982, including 680-29-1. Sunone cultivation began with a purple-stem landrace, and one entity with a purple stem, broad leaves, and six round, palmately compound leaves chosen at the production site. It was labeled 78135 and selected as the first rot-resistant root variant in 1996. In 2000, after adaptation testing, it was registered as the new variety, Sunone. The aerial part of Sunone has anthocyanin-rich purple stems, inflorescence, round and simple berries, and stolon roots. The berries and autumn leaves of Sunone are red. The leaflets are broad and elliptical, and the root color is cream. The average stem length, stem diameter, leaf length, leaf width, palmate cleaves, and number of leaflets are 35.4 cm, 7.5 mm, 15.7 cm, 6.8 cm, 4.5, and 24.4, respectively. The three-year-old main root average length, diameter, and root weight are 8.5 cm, 25.3 mm, and 84.1 g, respectively. For the quality of red ginseng, the average rates of heaven- and earth-grade ginseng are 1.9% and 6.8%, respectively. It has 61.1% resistance against root rot, but that depends on its age (Figure 2) [91].

### 4.10. K-1

The cultivation of K-1, one of the superior cultivars of *P. ginseng*, began in 1984. It is perfect for making red ginseng due to its good root shape, and it also has productive lateral roots and high disease resistance. K-1 is widely cultivated in many ginseng planting areas, including Chuncheon, Buyeo, and Gochang [1]. The growth characteristics of the roots in five-year-old K-1 plants begun by direct seeding are the main root length, diameter, and root weight of 10.6 cm, 20.0 mm, and 37.1 g, respectively (Figure 2) [1,96].

### 4.11. G-1

A new cultivar of *P. ginseng*, G-1, with different yield and ginsenoside content was developed in 2012. The morphological features of G-1 are violet stems that are stronger than Chunpoong, short flower stalks, budding later than Sunpoong, and red berries. The G-1 root appearance, disease resistance, and ginsenoside content were also analyzed (Figure 2) [97].

### 4.12. Kowon

In recent times, plant breeders have focused on developing new ginseng cultivars with higher resistance to biotic and abiotic stress. In 1999, seeds were collected from a farm in Suwon, Korea. After four years, good samples were screened for further evaluation, and from 2003 to 2007 cultivation and propagation characteristics were checked. In the elite line G03136-3, different yield trials were done from 2008 to 2010, and then the line was assigned the name Korea No. 1. After local adaptability tests from 2011 to 2013, Korea No. 1 was much-admired for its resistance to *Alternaria* blight and high yield. In November 2013, it was registered as a new cultivar named Kowon. The Kowon cultivar has broad green elliptical leaves with plain leaflets, yellowish-green stems, red fruits, and cream-colored roots. The main roots are cylindrical and intermediate in size compared with Yunpoong and Chunpoong. The growth characteristics of four-year-old Kowon ginseng are reflected by stem length, number of stems per plant, leaf length, leaf width, and number of leaves of 31.3 cm, 1.1, 17.5 cm, 7.3 cm, and 25, respectively. The root length, fresh weight, and diameter are 27.7 cm, 46.8 g, and 4.6 cm, respectively (Figure 2) [98].

### 4.13. Summary of Panax ginseng Cultivars in Korea

The preceding *P. ginseng* cultivars are well-developed cultivars from local landraces. The main purposes for breeding the different cultivars from 1970 to the late 1990s were high yield, quality of red ginseng, physical properties, high ginsenoside content, and disease resistance. However, due to increasing interest in red ginseng (for its pharmacological properties), attention has moved to develop cultivars resistant to high temperatures and with a high content of non-saponin bioactive components. Ginseng is a half-shade medicinal plant, and its growth can be reduced by intense light and high temperature [97,98,99]. To overcome these environmental problems and improve various characteristics related to ginsenoside production, several cultivars have been produced. Most of those cultivars were produced from Chunpoong and Yunpoong, which already have good morphology and high yield. Cultivars such as Chunpoong and Gumpoong are highly resistant to rust rot disease, Sunone is highly resistant to root rot disease, and Kowon is resistant to the *Alternaria* blight. The Sunun cultivar was the first to develop strong blistering on the surface of its leaflets. A high content of Aroma 1 is produced by the Sunhyang cultivar. Gopoong is mostly used for red ginseng products, and Yunpoong produces high root yields. Chunpoong, Yunpoong, Sunpoong, and Gumpoong are all good, reliable cultivars for red ginseng quality. 

## 5. *Panax ginseng* Cultivars in China

### 5.1. Jilin Huangguo Renshen 

**Figure 3:**
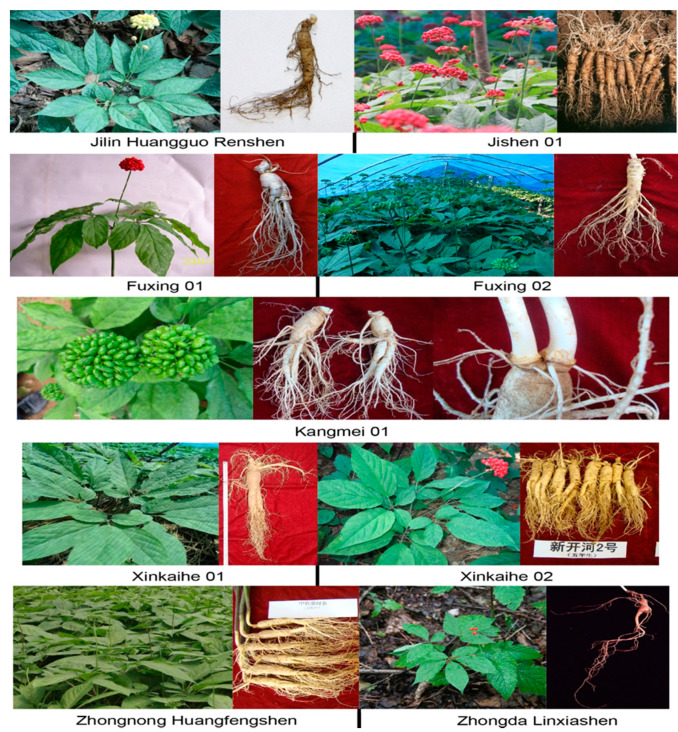
*P. ginseng* cultivars in China. Highly improved cultivars from elite lines having various characters regarding medicinal purposes.

A mutant ginseng plant with yellow mature fruit and green stems was discovered in Fusong County, Jilin province, in 1959. Subsequently, this plant was self-fertilized and propagated for several generations. The offspring showed stable heritability of yellow berries and green stems. Yellow-berry ginseng is a homozygous recessive mutant of red-berry ginseng. Comparisons between yellow- and red-berry ginseng have been conducted on agronomic traits, production, photosynthetic rate, and chemical constituents, including saponin. Very few differences are observed between them, but yellow-berry ginseng has significantly higher ginsenoside content and photosynthetic rate than red-berry ginseng (Figure 3) [100]. Based on its high ginsenoside content and yellow fruit, this population was kept continuously for self-fertilization for three generations. The selected group showed strong genetic stability in fruit color and ginsenoside content. Regional and production experiments were performed from 1981 to 1996, and it was finally registered as Jilin Huangguo Renshen in 1998.

### 5.2. Jishen 01 

To meet the requirements of ginseng growers in the eighteenth century, Jishen 01 was bred for its high production and good root shape. Originally, four inbred lines from the Ji’an region were selected to test eleven traits (yield, root weight, seedling survival, root length, main root length, root diameter, rhizome length, stem height, stem diameter, leaf length, and leaf width) (Figure 3). The inbred line G2, which showed the highest yield and excellent commodity characteristics, acquired the highest score in a comprehensive evaluation. In regional and production experiments, the yield of G2 was 15% higher, on average, than the local control population [101,102]. Jishen 01, which grows well in the Jilin, Liaoning, and Heilongjiang provinces, has heavy root weight, a long main root, good root shape, and a broad adaptation area. 

### 5.3. Fuxing 01

Fuxing 01 offers high yield, strong comprehensive resistance, short main root length, high root diameter, short rhizome length, large stem scar, and many fibrous roots and was registered in 2009. Fuxing 01 is suitable for growing in regions with an altitude lower than 1000 meters and 90–130 frost-free days. This cultivar was selected from the landrace Damaya, which formed in the specific geographical environment and climatic conditions of the Fusong region. A Damaya population was purified at Fusong State Ginseng Farm from 1989 to 2002 and named FX01. Then, regional and production experiments were performed in Changbai, Fusong, and Dunhua from 2003 to 2008. The average yield of the FX01 group was 2.47 kg/m^2^ in a production experiment, which is 0.41 kg/m^2^ higher than the local control. Interestingly, the highest yields for each year were all observed at the Fusong experimental site. In 2009, FX01 passed the examination and was registered as Fuxing 01 (Figure 3).

### 5.4. Fuxing 02

Fuxing 02 was also selected from the landrace Damaya, but it differs from Fuxing 01. Fuxing 02 has a high yield and ginsenoside content, a long growth period, and long main root length. Originally, 2900 excellent ginseng plants were selected in 1983, each with a main root length of more than 8 cm. Each plant was self-fertilized in 1984, and only 521 plants were reserved after removing plants that were diseased, had bad growth status, or produced few seeds. The seeds from each plant were harvested and bred into an inbred line. The inbred line was discarded whenever more than 50% of plants in any generation had a main root length of less than 8 cm. After selection for three generations, twelve inbred lines remained, and comparisons between them were carried out from 1995 to 1999. Inbred line 16 showed the best performance on yield, ginsenoside content, main root length, and survival rate. In regional and production experiments, the average yield increment of line 16 was 10.26% and 10.65%, respectively, compared with the local control. In 2014, line 16 was registered as Fuxing 02 (Figure 3). 

### 5.5. Kangmei 01

Kangmei 01 is the first farmland ginseng cultivar in China. As deforestation has limited the availability of traditional shaded fields, planting ginseng in open farmland has increased in China. However, ginseng cultivars bred to grow in forests have shown poor adaptability to the very different soil and climatic conditions found in farmland. Therefore, ginseng cultivars adapted to farmland are needed. To breed a farmland ginseng cultivar, 7730 ginseng seedlings whose root diameter and root weight were more than 2.0 cm and 25 g, respectively, at three years old and more than 2.2 cm and 33 g, respectively, at four years old were selected from the Damaya population. These selected lines were transplanted to farmland, and 26 kg seeds were harvested in 1991 (Figure 3). Then, those ginseng seeds were sown on farmland, and variants were removed from the group. The group was propagated and purified for another two generations and then named DD1. The breeders found that plants in the DD1 group often developed more than two stems (multi-stem plants). Regional and production experiments of DD1 were performed from 2006 to 2010. For different experiment sites and years, the yield of DD1 increased by 13.16% to 19.20% compared to local controls. Additionally, the multi-stem rate of DD1 plants was higher than 50% when the investigation was carried out on four-year-old ginseng plants. Eventually, DD1 was registered in 2012 as Kangmei 01 with the characteristics of high yield, robust roots, multi-stem, and good adaptability to farmland. 

### 5.6. Xinkaihe 01 

Biantiao ginseng has a beautiful root, with a long main root and branch root, consistent root diameter along the main root, and humanoid shape with a round shoulder and neck. To produce Biantiao ginseng, special cultivation methods and germplasm resources are needed. Because of its complicated cultivation procedure and low success rate, the price of Biantiao ginseng is much higher than that of common ginseng. Xinkaihe 01 was bred to produce high-quality Biantiao ginseng with a higher success rate. An Ermaya population produced through artificial selection and specific environmental factors in the Xinkai river basin was selected as the original material for breeding. A total of 3788 individuals that met the criteria for Biantiao ginseng were selected from that Ermaya population to construct a Biantiao group. The Biantiao group was propagated for four generations in an isolated field, and unqualified individuals were removed. This group showed a significantly higher yield, main root length, and Biantiao ratio compared with the local control in regional and production experiments. In 2013, this Biantiao group was designated as the ginseng cultivar Xinkaihe 01 (Figure 3).

### 5.7. Xinkaihe 02 

The ginseng cultivar Xinkaihe 02 was bred from the same Ermaya population as Xinkaihe 01, but it has a shorter rhizome than Xinkaihe 01. The breeding procedure for Xinkaihe 02 was similar to that for Xinkaihe 01, and the new cultivar was registered in 2016. Xinkaihe 02 has a long main root and short rhizome and is suitable for the production of model red ginseng (Figure 3).

### 5.8. Zhongnong Huangfengshen

Continuous cropping obstacles are one of the most important problems in ginseng cultivation. After ginseng has grown in it, a field requires about 30 years to recover enough for ginseng to be replanted. Although many techniques have been applied to improve the soil after ginseng harvest, continuous cropping of ginseng on a field is still hindered by fallen fibrous roots, rotten roots, and severe yield loss [102]. Therefore, breeding a ginseng cultivar resistant to those continuous cropping obstacles has been proposed. To breed a ginseng cultivar suitable for continuous cropping, 3000 high-resistance ginseng plants were screened from the Baoquanshan population. This high-resistance group was propagated in a single field for three generations. In each generation, nearly 50% of the plants were dead, and the remaining plants were named group ZH01. In regional and production experiments, the yield of ZH01 was at least 98% higher than that of the local control when grown continuously on a ginseng field and at least 9.9% higher when grown in a forested field. Furthermore, ZH01 showed a more flourishing fibrous root than the control, especially when grown continuously in a single field. ZH01 was registered as Zhongnong Huangfengshen in 2016 (Figure 3). 

### 5.9. Zhongda Linxiashen

Zhongda Linxiashen is the only forest ginseng cultivar in China. Initially, 4700 forest ginseng plants with a beautiful humanoid root shape, long rhizome, and long fibrous roots were collected from the Changbai mountains. The seeds of those plants were harvested together and sown in an isolated field. The traits of the underground parts were investigated when the F1 plants were ten years old. The plants that did not achieve the breeding targets were discarded, and the seeds of the remaining plants were harvested. This procedure was repeated for four generations, and the elimination rates were 51.2%, 39.8%, 31.6%, and 25.5% for F1–F4, respectively (Figure 3). In regional and production experiments, the percentage of high-quality ginseng (higher than grade 2 according to the national standard, Identification and Grade Quality Standards of Wild Ginseng GB/T 18765-2015/2008) from this group was at least 35.6%, whereas the maximum percentage from the local control was 24.2%. This ginseng cultivar was designated as Zhongda Linxiashen and registered in 2016.

### 5.10. Summary of Panax ginseng Cultivars in China

Most ginseng cultivars (Jilin Huangguo Renshen, Jishen 01, Fuxing 01, Fuxing 02, Kangmei 01, Xinkaihe 01, and Xinkaihe 02) were bred in Fusong and Ji’an, which account for half of the area of ginseng cultivation in China. From 1960 until the 1990s, the major target traits in ginseng breeding were yield, root shape, and ginsenoside content. When deforestation created a shortage of fields suitable for ginseng cultivation in the late 1990s, breeders changed their focus to cultivars amenable to farmland and continuous cropping. As it takes about fifty years to breed a forest ginseng cultivar, only one forest ginseng cultivar is currently registered. Among the Chinese ginseng cultivars, Jishen 01, Fuxing 01, and Fuxing 02 are representative high yield cultivars. Xinkaihe 01 and Xinkaihe 02, with long main roots, were bred specifically for Biantiao ginseng production. Jilin Huangguo Renshen is the only cultivar bearing yellow fruit; the others all have red fruit. Kangmei 01 is suitable for farmland growing, and Zhongnong Huangfengshen is highly resistant to the obstacles common with continuous cropping. Zhongda Linxiashen has a beautiful root shape and is used to produce high-quality wild ginseng. 

## 6. Conclusion and Future Prospects

The main goal of this review has been to profile all Korean and Chinese *P. ginseng* cultivars in terms of their good characteristics. The major purpose of *P. ginseng* cultivar selection has been to elaborate those with the quality of red ginseng, good physical properties, resistance to disease, resistance to root rot, high yield, and high saponin content. Also, selected cultivars should be superior to a previous cultivar or its landraces. Accordingly, different cultivars have been developed to overcome problems commonly faced during ginseng root growth, control biotic and abiotic stress, and increase useful bioactive components. Therefore, this review will enable readers to select ginseng cultivars suitable for a healthy life. In the future, the characteristics of existing cultivars can continue to be improved, and new cultivars of other *Panax* species can be developed by inter- or intraspecific hybridization.

## Figures and Tables

**Table 1:** Secondary metabolites produced by ginseng.

No	Compounds	Medicinal Properties	References
1.	Ginsenoside	Anti-cancer, Anti-diabetes, Anti-inflammation, Hepatoprotection, Anti-aging, Anti-oxidative	[30,31,32,33,34]
2.	Phytosterol (Stigmasterol and β-sterol)	Lower the cholesterol level	[35,36]
3.	Sesquiterpenes (β-elemene and β-selinene)	-	[35,37,38]
4.	Flavonoids (Kaempferol)	Anti-oxidant, Hepatoprotective, Anti-cancer, Anti-inflammatory, Anti-viral	[35,39]
5.	Polyacetylenes (Panaxynol, Ginsenoyne A)	Possess Anti-tumor properties	[35,40]
6.	Alkaloids (Fumarine, Girinimbin)	-	[35,41]
7.	Phenolic Compounds (Elemicin, Dauricine, Maltol)	Anti-tumor, Anti-oxidant, Anti-inflammatory	[3,35,42,43]

**Table 2:** The list of seventeen *Panax* species in the world.

No.	Ginseng Species	Common Names	References
1	*P. ginseng*	Korean ginseng	[23,24,44,45,46,47,48] https://www.biodiversitylibrary.org/
2	*P. quinquefolius*	American ginseng	[45,49,50], https://www.biodiversitylibrary.org/, http://powo.science.kew.org/
3	*P. notoginseng*	Chinese ginseng	[45,47], http://powo.science.kew.org/, https://www.gbif.org/
4	*P. japonicas*	Japanese ginseng	[45,51,52], http://powo.science.kew.org/
5	*P. omeiensis*	Omei ginseng	[43,53]
6	*P. pseudoginseng*	Himalayan ginseng	[45,54,55] http://powo.science.kew.org/
7	*P. assamicus*	N/A	[45,56] http://powo.science.kew.org/
8	*P. shangianus*	N/A	[57,58]
9	*P. sinensis*	N/A	[43,57]
10	*P. stipuleanatus*	Pingpien ginseng	[43,56], https://www.gbif.org/, http://powo.science.kew.org/, https://www.ipni.org/
11	*P. trifolius*	Dwarf ginseng	[45,59], https://www.biodiversitylibrary.org/
12	*P. variabilis*	N/A	[56,57]
13	*P. vietnamensis*	Vietnamese ginseng	[57,58], https://www.gbif.org/, http://powo.science.kew.org/, https://www.ipni.org/
14	*P. wangianus*	Narrow-leaved pseudoginseng	[43,57], http://powo.science.kew.org/, https://www.gbif.org/, https://www.ipni.org/
15	*P. bipinnatifidus*	Feather-leaf bamboo ginseng	[45,60], http://powo.science.kew.org/
16	*P. sokpayensis*	N/A	[60], http://powo.science.kew.org/
17	*P. zingiberensis*	Ginger ginseng	[43,56,57,58], http://powo.science.kew.org/, https://www.ipni.org/

**Table 3:** Some of the ginsenoside structures and its medicinal properties..

No.	Species	Chemical Structure	Medicinal Properties	References
1.	*Pg*, *Pq, Pn, Pj*	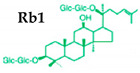	Anti-arthritic, Central inhibition and mental stability, Promoting protein synthesis, Cold tolerance	[2,67,68,69,70]
		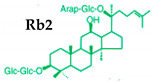	Anti-nociception, Immune modulating, Wound healing, Inhibits angiogenesis	[2,3,71]
		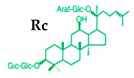	Anti-inflammatory, Anti-arthritic, Anti-gastritis	[2,72,73]
		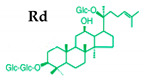	Anti-hypertension, Anti-oxidant, Free radical scavenger, Immunological adjuvant activity	[2,74,75]
		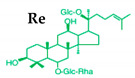	Non-genomic effects in endothelial cells, Enhance Angiogenic, Anti-diabetes	[2,76,77]
		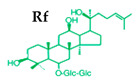	Anti-nociception, Pain inhibition, Inhibition of lipid peroxidation, Physiological saline control	[2,78,79]
		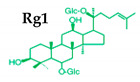	Anti-coagulation, Anti-platelet aggregation, Prolonged clotting time, Anti-diabetes	[2,77,80]
		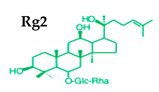	Anti-apoptosis, Anti-oxidation, Neuroprotective, Treatment for Alzheimer’s disease	[2,81]
2.	*Pg*, *Pq, Pj*	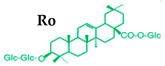	Inhibit the replication of HIV-1, Reduced ischemic brain injury	[2,82,83]
3.	*Pq, Pn*	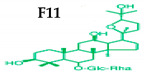	Assists memory improvement neuroprotective	[2,29]

Note: *Panax ginseng (Pg), Panax quinquefolius (Pq), Panax notoginseng (Pn), Panax japonicus (Pj).*
